# Microbial changes from bariatric surgery alters glucose-dependent insulinotropic polypeptide and prevents fatty liver disease

**DOI:** 10.1080/19490976.2023.2167170

**Published:** 2023-02-02

**Authors:** Tien S. Dong, William Katzka, Julianne C. Yang, Candace Chang, Nerea Arias-Jayo, Venu Lagishetty, Anna Balioukova, Yijun Chen, Erik Dutson, Zhaoping Li, Emeran A. Mayer, Joseph R. Pisegna, Claudia Sanmiguel, Jonathan P. Jacobs

**Affiliations:** a UCLA Center for Human Nutrition, University of California, Los Angeles, California, USA; b David Geffen School of Medicine, University of California, Los Angeles, California, USA; c G. Oppenheimer Center for Neurobiology of Stress and Resilience, University of California, Los Angeles, California, USA; d UCLA Microbiome Center, David Geffen School of Medicine at UCLA, Los Angeles, California, USA; e Division of Gastroenterology, Hepatology and Parenteral Nutrition, VA Greater Los Angeles Healthcare System, Los Angeles, California, USA; f Department of Surgery, UCLA Center for Obesity and METabolic Health (COMET), Los Angeles, California, USA; g The Vatche and Tamar Manoukian Division of Digestive Diseases, Department of Medicine, David Geffen School of Medicine at UCLA, Los Angeles, CA, USA

**Keywords:** Akkermansia, GIP, NAFLD, bariatric surgery, sleeve gastrectomy

## Abstract

Bariatric surgery remains a potent therapy for nonalcoholic fatty liver disease (NAFLD), but its inherent risk and eligibility requirement limit its adoption. Therefore, understanding how bariatric surgery improves NAFLD is paramount to developing novel therapeutics. Here, we show that the microbiome changes induced by sleeve gastrectomy (SG) reduce glucose-dependent insulinotropic polypeptide (GIP) signaling and confer resistance against diet-induced obesity (DIO) and NAFLD. We examined a cohort of NALFD patients undergoing SG and evaluated their microbiome, serum metabolites, and GI hormones. We observed significant changes in *Bacteroides*, lipid-related metabolites, and reduction in GIP. To examine if the changes in the microbiome were causally related to NAFLD, we performed fecal microbial transplants in antibiotic-treated mice from patients before and after their surgery who had significant weight loss and improvement of their NAFLD. Mice transplanted with the microbiome of patients after bariatric surgery were more resistant to DIO and NAFLD development compared to mice transplanted with the microbiome of patients before surgery. This resistance to DIO and NAFLD was also associated with a reduction in GIP levels in mice with post-bariatric microbiome. We further show that the reduction in GIP was related to higher levels of *Akkermansia* and differing levels of indolepropionate, bacteria-derived tryptophan-related metabolite. Overall, this is one of the few studies showing that GIP signaling is altered by the gut microbiome, and it supports that the positive effect of bariatric surgery on NAFLD is in part due to microbiome changes.

## Graphical abstract

**Figure:**
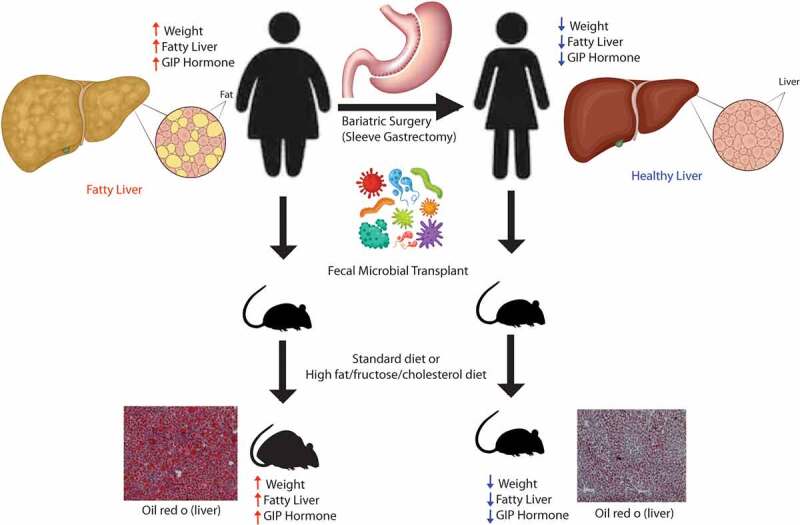

## Introduction

Obesity affects one out of every four Americans, and nonalcoholic fatty liver disease (NAFLD) affects almost 40–50% of obese patients^1^. NAFLD is estimated to affect up to 30–50% of all obese patients.^2^ There are many factors that contribute to the development of NAFLD, which include genetics, lipid metabolism, diet, and host metabolism. Despite the rising tide of NAFLD incidence, there are currently no approved medications for the treatment or prevention of NAFLD. While diet and exercise remain pivotal to the treatment of NAFLD, only 10–15% of patients are able to reach and sustain sufficient weight loss to affect NAFLD progression.^3^ One of the most effective long-term therapies for obesity and NAFLD has been bariatric surgery.^4^ However, not every NAFLD patient is a candidate for bariatric surgery, and not every eligible patient is willing to undergo surgery for weight loss. Therefore, continued research in this field of medical therapy is paramount to the development of novel therapeutics.

One area that has shown promise in the field of NAFLD is the gut microbiome. Over the last decade, the gut microbiome has been shown to play a pivotal role in the development of obesity, insulin resistance, NAFLD, and liver fibrosis.^5–9^ Germ-free mice have lower body fat and are more resistant to obesity than conventionally housed mice.^10^ Using fecal microbial transplants, the obesity phenotype has been shown to be transferable via the gut microbiome.^11^ Similar transfer experiments were done in obese patients undergoing bariatric surgery.^12^ Yet, the exact mechanism by which the gut microbiome modulates obesity and NAFLD is still unclear. In our study examining human data, we have shown specific bacterial taxa associated with hepatic steatosis,^13^ NAFLD-related fibrosis,^14^ and weight loss.^15^ While many studies have shown strong associations with the intestinal microbiome and NAFLD, few have shown causal links. *Therefore, the purpose of our study is to examine the mechanism by which microbiome changes induced by bariatric surgery can cause alterations in weight and NAFLD development*. In our current study, we have shown through microbial transfer into antibiotic-treated mice that the gut microbiome changes induced by bariatric surgery impart resistance to weight gain and NAFLD through altered glucose-dependent insulinotropic polypeptide (GIP) hormone signaling.

GIP and glucagon-like peptide (GLP-1) are two incretin hormones secreted by the enteroendocrine system. However, unlike GLP-1, GIP is less studied and has not yet become a pharmacological therapy for the treatment of obesity or NAFLD. GIP plays an important role in lipid metabolism, satiety and insulin sensitivity.^16^ We show in our cohort of NAFLD patients undergoing bariatric surgery that GIP hormone levels decrease significantly with sleeve gastrectomy and weight loss. In our microbiome transfer experiments, mice with the microbiome of prebariatric surgery patients (i.e., obese) had higher levels of GIP hormone and worse NAFLD than mice with the microbiome of post-bariatric surgery patients (i.e., leaner).

## Results

### Sleeve gastrectomy leads to significant reduction in weight and inflammatory markers

**Figure 1.**
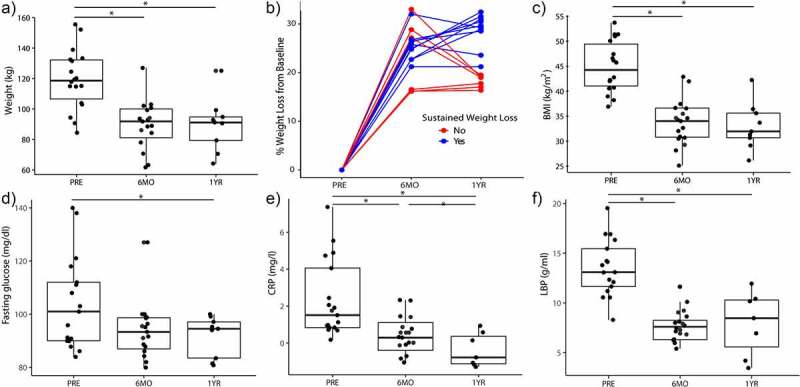
Bariatric surgery in humans leads to reduction in weight, insulin resistance, and inflammatory markers. (A) Weight change of patients undergoing sleeve gastrectomy at 6-month and 1-year post-sleeve gastrectomy. (B) Percent weight change from baseline at 6-month and 1-year post-surgery, colored by those with sustained weight loss (i.e., at least 20% weight loss). Also shown, is (C) BMI, (D) fasting glucose, (E) C-reactive protein (CRP), and (F) lipopolysaccharide binding protein (LBP) over time. *indicates *P*-values<0.05. *N* = 18.

Mirroring larger and previously published studies on bariatric surgery, we evaluated a small cohort of NAFLD patients undergoing bariatric surgery and collected their stools for our mouse transfer experiment. We examined a cohort of morbidly obese females (n = 18), all of whom had NAFLD, undergoing sleeve gastrectomy. The average age of our cohort was 37.1 ± 9.4 years old. The average weight and BMI at baseline were 118.5 ± 18.8 kg and 44.7 ± 4.9, respectively. The ethnicity of patients was predominantly non-Hispanic White (44.4%) followed by Hispanic (38.9%) (Supplemental Table S1) There was significant weight loss at 6 months (118.5 kg ± 18.8 vs 89.7 kg ± 16.9, *P* < 0.001). Of the 18 patients, 12 had sustained weight loss of at least 20% of baseline bodyweight at 1 year. Associated with weight loss, there was a significant reduction in fasting glucose and inflammatory markers, such as C-reactive protein (CRP) and lipopolysaccharide-binding protein (LBP) (Figure 1) at 6 months.

*Gut Microbiome Predicts Weight Loss and Sleeve Gastrectomy Leads to Significant Changes in Microbiome and Serum Metabolites*

**Figure 2.**
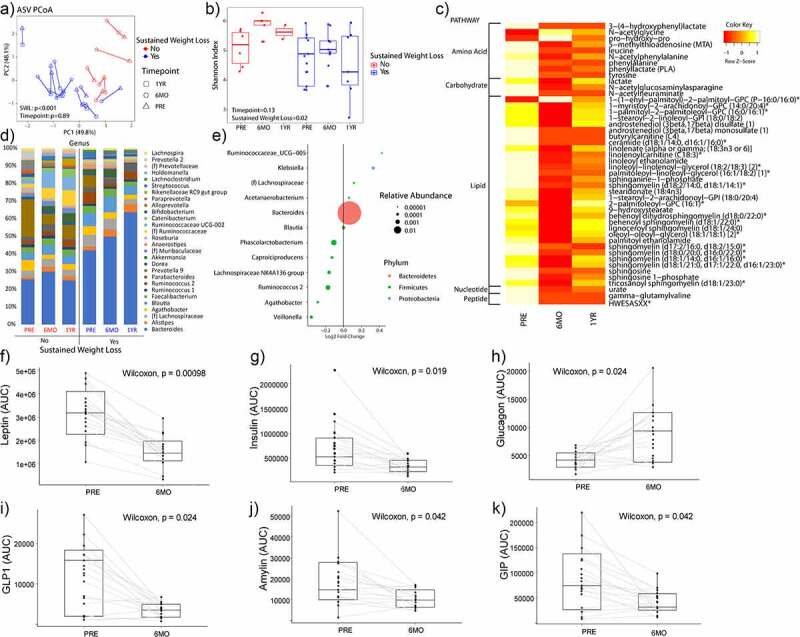
Microbiome, serum metabolite, and GI hormone changes with bariatric surgery. (A) Principal coordinate analysis plot (i.e., beta diversity). Each dot represents a patient sample and it is colored by sustained weight loss with shapes indicating what time point the sample was collected. Lines connect an individual patient sample over time. (B) Alpha diversity as measured by Shannon Index. (C) Heatmap showing all the differentially abundant serum metabolites grouped by super pathways. (D) Taxonomic summary plots of genera with at least a relative abundance of ≥1%. (E) Differential abundance testing by MaAsLin showing bacteria genera that are different at 6 months as compared to baseline for patients adjusting for sustained weight loss (SWL) with patient ID as a random effect. (F-K) For the GI hormones, patients were given a standard meal and blood at fasting, 30 minutes, 60 minutes, and 90 minutes were sampled for various GI hormones. Circulating GI hormone was then measured across time and the area under the curve (AUC) for each hormone at baseline and at 6-months post-sleeve gastrectomy is shown for (F) leptin, (G) insulin, (H) glucagon, (I) glucagon-like peptide 1 (GLP1), (J) amylin, and (K) glucose-dependent insulinotropic polypeptide (GIP). *N* = 18.

Patients who had sustained weight loss of at least 20% from their baseline weight had significantly different microbiomes at baseline as evaluated through beta diversity (p < .001) at 6 months (Figure 2). Patients with sustained weight loss also had significantly lower alpha diversity at baseline and at follow-up (*P* = 0.02). Taxonomic plots by genus showed that patients with sustained weight loss (SWL) had more *Bacteroides* at baseline and that the relative abundance of *Bacteroides* increased with time. However, patients without sustained weight loss had lower relative abundance of *Bacteroides* at baseline, and their levels did not change over time.

There were no significant differences in either beta or alpha diversity between patients’ samples before vs. after surgery. Microbiome analysis between groups adjusting for sustained weight loss and patients showed that patients had 12 genera that were altered after bariatric surgery. These included *Ruminococcaceae UCG-005, Klebsiella*, (f) *Lachnospiraceae*, *Acetanaerobacterium, Bacteroides, Blautia, Phascolarctobacterium, Capriociproducens, Lachnospiraceae NK4A136 group, Ruminococcus 2, Agathobacter, Veillonella* (*q*-value <0.05). The genus with the largest relative abundance was *Bacteroides* (22.5% at baseline in patients without sustained weight loss and 36.6% at baseline in patients with sustained weight loss) and it was increased after sleeve gastrectomy (25.0% at 6-month post-surgery in patients without sustained weight loss and 42.8% at 6-month post-surgery in patients with sustained weight loss).

In addition to 16S sequencing, fasting blood serum was analyzed for metabolites using an untargeted metabolomic platform. After adjusting for multiple hypothesis testing, 46 metabolites were found to be different at 6-month post-surgery as compared to baseline values. Thirty-one of the 46 metabolites were involved in lipid metabolism and nine were related to amino acid metabolism. Figure 2c shows each differentially abundant metabolite and their relative amount across the three timepoints.

#### Sleeve gastrectomy leads to significant alteration of circulating gastrointestinal hormones

Similar to microbiome and serum metabolite changes, sleeve gastrectomy also led to significant changes in circulating gastrointestinal hormones. Eleven different GI hormones were tested and of those six were altered by sleeve gastrectomy (figure 2f-K). Leptin (*P* < 0.001), insulin (*P* = 0.019), C-peptide (*P* = 0.02), glucagon-like peptide-1 (GLP-1) (*P* = 0.024), amylin (*P*p = 0.042), and GIP (*P* = 0.042) were reduced at 6-month post-sleeve gastrectomy, while glucagon (*P* = 0.024) was increased at 6-months. There was no difference at 6-months as compared to the baseline values for IL-6, MCP-1, pancreatic polypeptide (PP), peptide YY (PYY), and ghrelin (Supplemental Figure S1).

#### Microbiome changes induced by bariatric surgery prevents diet-induced obesity and NAFLD development

**Figure 3.**
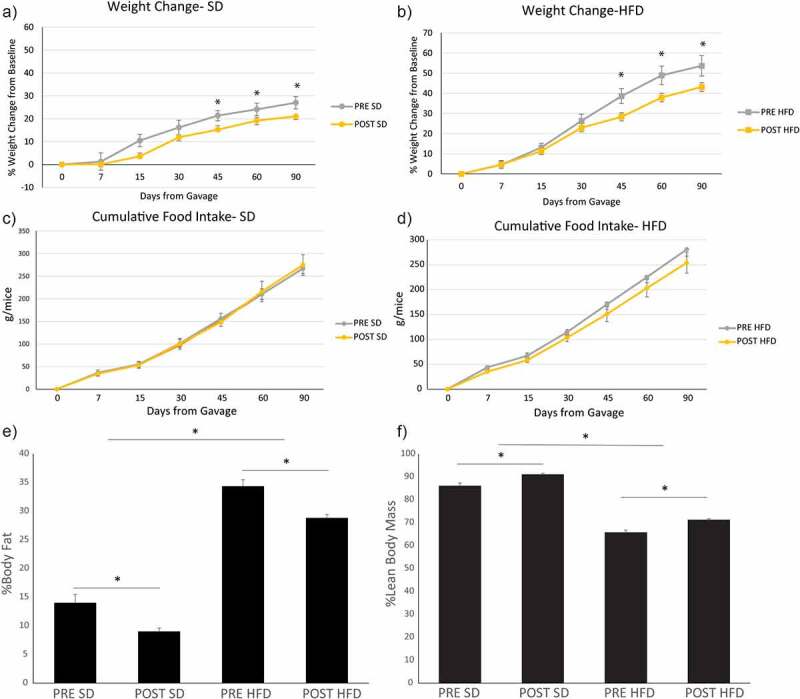
Bariatric surgery-induced microbiome changes protect against diet-induced obesity. Microbiome from donors before bariatric surgery (PRE) and after bariatric surgery (POST) were transplanted into antibiotic treated mice. Mice were then placed on either a standard diet (SD) or a diet high in fat, fructose, and cholesterol (HFD) for 90 days. Weight change of mice of the PRE and POST group on a (A) SD or a (B) HFD. Cumulative food intake of mice on a (C) SD or a (D) HFD. Body composition of the mice by EchoMRI as shown as (E) percent body fat or as (F) percent lean body mass. *indicates *P*-values<0.05. *N* = 16 per group.

To prove a causal link between the microbiome changes induced by sleeve gastrectomy and NAFLD, we performed fecal microbial transplants from human donors into antibiotic-treated mice and placed the mice on either a standard diet (SD) or a diet high in fat, fructose, and cholesterol (HFD) for 90 days. Because of the important role that the immune system plays in NAFLD development and progression, we opted to use antibiotic treated mice instead of germ-free mice, as germ-free mice have an altered immune system as compared to SPF mice.^17^ All donors had sustained weight loss of at least 20% reduction in body weight at 6 months. All donors also had NAFLD at the baseline that resolved 6 months after bariatric surgery. 16S sequencing and beta diversity testing showed that the mice were most similar to each other and their human donor (Supplemental Figure S2) and that the mice had at least 70% of their human donor genera present within their newly established microbiome. Antibiotic treated mice transplanted with baseline donor stools (i.e., PRE) gained significantly more weight while on SD or HFD as compared to antibiotic-treated mice transplanted with donor stool 6 months after sleeve gastrectomy (i.e., POST) (Figure 3). There were no significant differences in cumulative food intake in either group. The difference in weight gain was due to differences in fat percentage as measured by echoMRI. The POST group had significantly less percent body fat and higher percent lean body mass both on SD or HFD as the PRE group (*P* < 0.05). While there was a difference in weight gain, there were no differences in glucose tolerance testing or serum cholesterol while on a HFD (Supplemental Figure S3). The POST group did have improved glucose tolerance testing and serum total cholesterol and low-density lipoprotein/very low-density lipoprotein (LDL/VLDL) as compared to the PRE group but only while on a SD.

**Figure 4.**
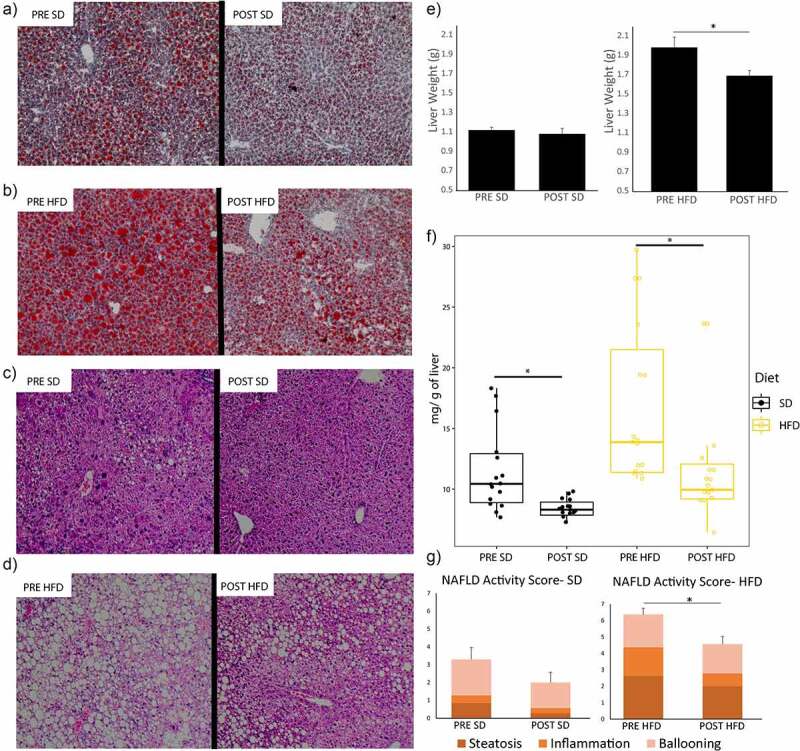
Bariatric surgery induced microbiome changes protect against NAFLD development. (A-B) Characteristic oil red o staining of liver section of mice receiving microbiome from patients before surgery (PRE) and 6 months after bariatric surgery (POST) on either a standard diet (SD) or a diet high in fat, fructose, and cholesterol (HFD). (C-D) Typical H&E staining of liver sections of mice by donor group (PRE vs POST) and by diet (SD vs HFD). (E) Excised liver weights of mice by donor group and diet. (F) Triglyceride quantification of liver sections. (G) NAFLD activity score of H&E-stained liver sections. *N* = 16 per group.

Similar to the findings on weight, the PRE group had significantly elevated hepatic steatosis while on SD and HFD as compared to the POST group both by oil red o staining (representative slides are shown in Figure 4 and quantification by ImageJ is shown in Supplemental Figure S4) and by triglyceride content (*P* < 0.05) (Figure 4). The weight of the liver was significantly lower in the POST HFD group as compared to the PRE HFD group (P < 0.05). The hematoxylin and eosin–stained liver sections of these mice were reviewed by an independent pathologist, and the NAFLD activity score was higher in the PRE group as compared to the POST only while on a HFD (*P* < 0.05).

#### Microbiome regulates GIP release potentially through shifts of Akkermansia and metabolites

**Figure 5.**
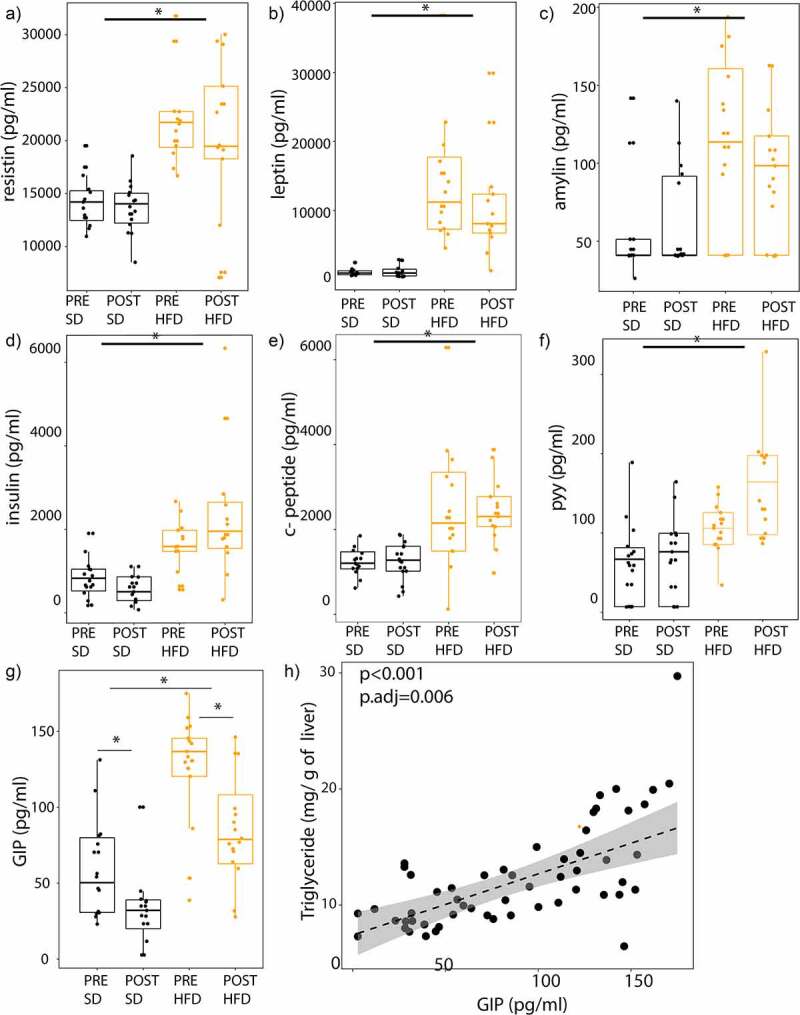
GIP is altered by the microbiome and diet and is associated with NAFLD severity. Serum samples of mice were collected after a 9-hour fast. Hormone profile is shown for (A) resistin, (B) leptin, (C) amylin, (D) insulin, (E) C-peptide, (F) PYY, and (G) GIP colored by diet (SD vs HFD) and by donor group (PRE vs POST). (H) Scatter plot showing the relationship between GIP and hepatic triglyceride content. Unadjusted and adjusted *P*-values adjusting for diet and donor group are shown. *indicates *P*-value<0.05. *N* = 16 per group, 64 mice total.

We then examined if these groups of mice had differing levels of gastrointestinal (GI) hormone expression. At the end of the 90 days, mice were fasted overnight, and blood was collected for GI hormone testing. While mice on a HFD had higher levels of resistin, leptin, amylin, insulin, C-peptide, and PYY hormone (*P* < 0.05), there were no differences in levels of these hormones between the PRE vs POST groups on either diet (Figure 5). The only hormone that was altered by the microbiome was GIP. The mice on a HFD had higher levels of GIP than mice on a SD (*P* < 0.001). Additionally, mice in the POST group had lower levels of GIP than mice in the PRE group both on a SD (*P* = 0.04) and an HFD (*P* = 0.02) (Figure 5). There were no significant differences in diet or microbiome donor group for any other GI hormones tested (Supplemental Figure S5). Using a general linear model, we observed that GIP levels were independently associated with hepatic triglyceride levels adjusting for the diet of the mice and whether they received stools from donors before or after surgery (i.e., donor group) (*P*.adj = 0.006) (Figure 5h).

**Figure 6.**
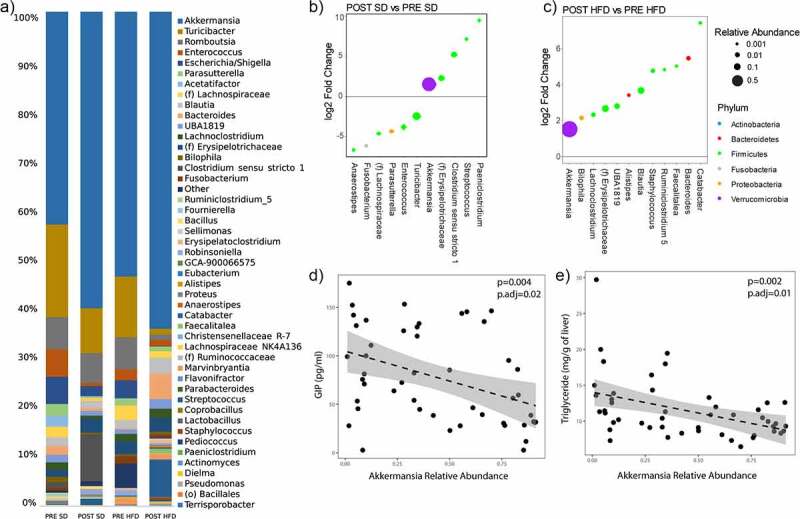
Mucosal *Akkermansia* is associated with the POST donor microbiome group and is negatively associated with hepatic triglyceride content and GIP levels. (A) Taxonomic plots of proximal small intestine mucosa-associated microbiome by donor group and diet. Genera shown are those with at least 0.1% relative abundance. (B-C) Differential abundance testing by DESeq2 between POST SD vs PRE SD and POST HFD vs PRE HFD. All bacteria shown have *q*-value <0.05. (D-E) Scatter plot showing the relationship between *Akkermansia* relative abundance and GIP level and hepatic triglyceride content, respectively. *P*-values listed are both unadjusted and adjusted for diet and donor group. *N* = 16 per group, 64 mice total.

Because GIP is expressed predominantly in the proximal small bowel, we then analyzed the composition of the microbiome in the proximal small intestine of these mice using 16S sequencing (Figure 6), specifically examining the mucosa-associated microbiome. The major genus that made up the proximal small bowel mucosa microbiome was *Akkermansia*. From the taxonomic plots, we see that *Akkermansia* increased in the POST group as compared to the PRE group. Differential abundance testing showed this relationship to be true in both the SD (Figure 6b) and the HFD group (Figure 6c) (*q*-value <0.05). An unidentified genus belonging to the family *Erysipelotrichaceae* was also elevated in the POST group on both HFD and SD. No other genera were differentially abundant between the PRE and POST groups adjusting for diet. *Bacteroides*, was only elevated in the POST group while on a HFD alone. Of the two genera that were different in the POST group as compared to the PRE group on both diets, only *Akkermansia* was associated with GIP level independent of diet and donor group (*P*.adj = 0.02). We see that *Akkermansia* had a negative association with GIP levels (Figure 6d). Furthermore, when we analyzed whether *Akkermansia* was associated with NAFLD, we saw that *Akkermansia* was negatively associated with hepatic triglyceride, independent of diet and donor group (*P*.adj = 0.01) (Figure 6d). *Bacteroides* was not associated with GIP levels or hepatic triglyceride. *Post-hoc* analysis of *Akkermansia* levels in the donor stool showed that the donors’ average relative abundance of *Akkermansia* in their stool was 0.15% before surgery and the average relative abundance of *Akkermansia* in their stool after surgery was 13.6% (*P* = 0.12).

**Figure 7.**
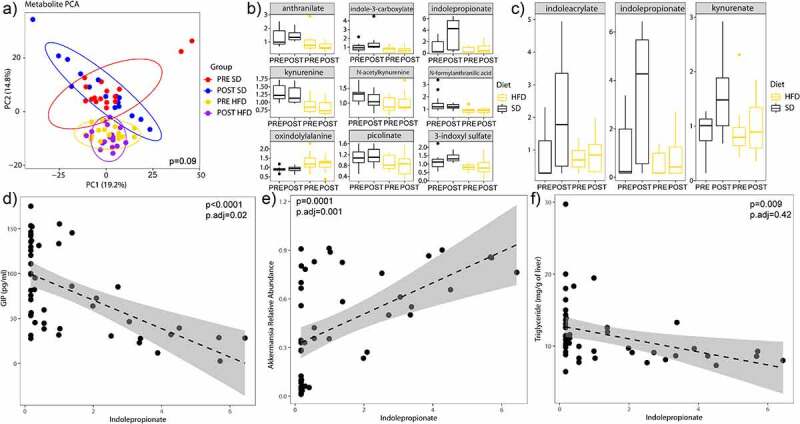
Indolepropionate is positively associated with *Akkermansia* and negatively associated with GIP. (A) Principal component analysis plot of portal vein metabolites by diet and donor group. Ellipses shown are 95% confidence intervals. (B) Tryptophan-related metabolites that are different by diet when adjusting for donor group (*P*-values<0.05) (data represented is a relative scale using a median-scale normalization method). (C) Tryptophan-related metabolites that are different by donor group adjusting for diet (*P*-values<0.05) (data represented is a relative scale using a median-scale normalization method). Scatter plot showing relationship between indolepropionate and (D) GIP, (E) *Akkermansia*, and (F) hepatic triglyceride content. *P*-values shown are unadjusted and adjusted for diet and donor group. *N* = 16 per group, 64 mice total.

In order to examine the potential function of these microbiota changes and how they may be related to NAFLD, we also processed the portal blood for circulating metabolites. Portal blood was selected for metabolite analysis to identify bacterial metabolites that could cross the epithelial barrier and thereby have the greatest potential to modulate gastrointestinal hormone production and directly affect NAFLD development. Because tryptophan-related metabolites are highly dependent on the microbiome^18^ and have been shown to be directly related to obesity,^19^ we focused our analysis on tryptophan-related metabolites. The full list of the 21 metabolites tested is shown in Supplemental Table S3. From the principal component analysis, we see that the majority of metabolite differences were due to whether the mice were on a SD or a HFD (Figure 7a). Nine tryptophan-related metabolites were associated with diet independent of donor group (*P* < 0.05) (Figure 7b). The metabolites that were higher in SD were anthranilate, indole-3-carboxylate, indolepropionate, kynurenine, N-actylkynurenine, N-formylanthranillic acid, picolinate, and 3-indoxyl sulfate. Only the metabolite oxindolylalanine was lower in the SD group as compared to the HFD group. When adjusting for the diet, only three tryptophan-related metabolites were different in the POST group as compared to the PRE group: indoleacrylate indolepropionate, and kynurenate (*P* < 0.05) (Figure 7c). Of these metabolites, only indolepropionate was associated with GIP. Adjusting for diet and donor group, Indolepropionate was negatively associated with GIP (*P*.adj = 0.02) and positively associated with *Akkermansia* (*P*.adj = 0.001). Indolepropionate was also negatively associated with hepatic triglyceride content (unadjusted *P*-value = 0.009), but after adjusting for diet and donor groups, it was not significant (*P*.adj = 0.42).

## Discussion

Bariatric surgery has the best long-term outcome profile among treatments for obesity and NAFLD. While there are many factors that are responsible for weight loss in bariatric surgery patients, we show here in our study that the microbiome changes induced by bariatric surgery can alter weight gain and NAFLD development through changes in GIP signaling. Even though the main reason for our human study was to gain fecal samples before and after surgery for our mouse transfer experiment, our small cohort showed similar positive long-term effects of sleeve gastrectomy on obesity and NAFLD as previously published data on larger cohorts.^20^ Patients who underwent laparoscopic sleeve gastrectomy were able to achieve on average 25.4% of total body weight loss at 1 year. This was associated with a decrease in inflammatory markers, such as hs-CRP and LBP. Previous studies examining adipose tissue have shown that pro-inflammatory cytokines like IL-6 and tumor necrosis factor-α (TNF-α) expression are higher in adipose tissue of obese patients as compared to lean patients.^21,22^ In other prospective studies examining bariatric surgery, researchers have found that, with bariatric surgery and a decrease in adiposity, IL-6 and TNF-α decreases both in the serum and in the adipose tissue.^20^ Severe obesity is also marked by severe insulin resistance, leptin resistance, and elevated GIP levels.^23^ Here, we show that with a reduction in weight through bariatric surgery, insulin resistance is improved, and basal leptin and GIP levels are lowered, similar to larger human trials of bariatric surgery patients.^20^

While laparoscopic sleeve gastrectomy may not alter the GI tract as much as a Roux-en-Y gastric bypass, it is still able to cause significant long-term shifts in the gut microbiome. In our study, we saw that *Bacteroides* increased after sleeve gastrectomy and this increase was more pronounced in patients who had sustained weight loss. In studies examining the gut microbiome and obesity, *Bacteroides* has been shown to be negatively associated with obesity.^10,24,25^ Therefore, the increase in *Bacteroides* over time is in line with previously published microbial data.

To show that microbiome changes in bariatric surgery are causally related to NAFLD, we transplanted the microbiome of patients with NAFLD before and after bariatric surgery into antibiotic treated mice. The results show that the microbiome of patients before bariatric surgery increased weight and body fat gains as compared to the microbiome after surgery. These changes were also associated with worse NAFLD and higher levels of GIP irrespective of diet. In a landmark paper by Tremaroli *et al.*, they showed similar results of increased weight gain in germ-free mice transplanted with pre-bariatric microbiome compared to germ-free mice transplanted with post-bariatric microbiome while on a standard diet.^12^ In their study, there was no difference in food intake, respiratory quotient, or activity level in mice transplanted with controlled microbiome versus those transplanted with post-laparoscopic sleeve gastrectomy microbiome.^12^ Our study corroborates that finding. The exact mechanism by which the gut microbiome can cause obesity is still an active area of research. It is likely multifactorial with evidence of bile acid modulation, farnesoid X receptor signaling, short-chain fatty acid signaling, alterations in the brain–gut axis, and increased energy extraction.^26–28^ In our study, we propose that another mechanism is through alteration of such key GI hormones as GIP.

GIP is an incretin hormone released by K cells in the small intestine after the intake of either fat or glucose. Animal models from global knockout studies of GIPR and antagonism of GIPR have shown that a reduction of GIP signaling is protective against obesity and NAFLD.^29^ Mice with adipocyte-specific GIPR KO had lower hepatic triglyceride content and hepatic steatosis even while on a high-fat diet, and this change was associated with a significant reduction of the pro-inflammatory adipokine, IL-6.^30^ There is also evidence showing that GIP release leads to liver fat accumulation with consumption of foods with high glycemic index (i.e., simple sugars like sucrose) as compared to those with low glycemic index (i.e., complex sugars like isomaltulose).^31^ Faster digestion of sucrose and more proximal small intestine uptake of glucose led to higher levels of GIP release, while slower digestion of isomaltulose bypassed GIP release by K cells and stimulated GLP-1 release by L cells in the distal small intestine.^31^ Further evidence supporting the role of GIP in hepatic steatosis is shown in animal models chronically fed with a diet high in sucrose. Diets high in sucrose resulted in higher glucose absorption, higher GIP release, and higher hepatic fat accumulation as compared to diets high in isomaltulose. This difference was absent in mice with deletion of GIPR.^32^ This has led many to believe that GIP antagonism can be used as a potential target for NAFLD. This has been proven in several mouse models where GIP antagonism administration was shown to reduce steatosis and weight in mice with NAFLD.^33,34^

Because the majority of GIP is released in the proximal small intestine, we examined the microbial composition of the proximal small intestine mucosa to see which bacteria may be regulating GIP release, and we found that *Akkermansia* was negatively associated with GIP levels. *Akkermansia* is likely one of the most well-studied genera relating to both obesity and fatty liver disease.^35–37^ Several studies in humans, including our own, have shown that *Akkermansia* was negatively associated with obesity and NAFLD.^13,19,37–39^ Mechanisms by which *Akkermansia* is protective against obesity include restoration of gut epithelial function, changes in lipid metabolism, and regulation of the host immune system.^40^ In a small human clinical trial, *Akkermansia* supplementation led to improved weight and insulin resistance.^41^ In our study, we propose that *Akkermansia* may also be of benefit to NAFLD and obesity by inhibiting GIP release.

One way by which microbes communicate with the host is through alterations of metabolites. Similar to our previous work on tryptophan-related metabolites in obesity,^19^ we found that three tryptophan-related metabolites in the portal vein associated with microbiome changes independent of diet. Of those three, only indolepropionate was independently associated with GIP levels, with increasing levels of indolepropionate associated with lower levels of GIP. Indoles are created through the bacterial metabolism of tryptophan, and their production is heavily dependent on bacteria. A metabolite study demonstrated that indolepropionate was only detectable in the plasma of conventionally housed mice and not germ-free mice.^18^ One known function of indolepropionate is its ability to bind and activate Pregnane X receptor (PXR) which leads to improved intestinal barrier function and reduction of enterocyte tumor necrosis factor-α (TNF-α), two features that are abnormal in patients with NAFLD.^42^ Additionally, indolepropionate has strong correlations with human obesity and fatty liver disease. For example, in our previous research on obese females with food addiction, indolepropionate was negatively associated with obesity and food addiction while being positively correlated with *Akkermansia*.^19^ In a Finnish study, indoleproprionate was negatively associated with type 2 diabetes.^43^ Therefore, the positive correlation of indolepropionate with *Akkermansia* and negative association with GIP levels is consistent with previously published studies.

## Limitations of study

While this study does show a novel interaction between bariatric surgery-induced microbiome changes and GIP release, the human cohort is small and consists only of females, and so is potentially affected by sampling bias. Therefore, contradictory findings such as GLP-1 decreasing after sleeve gastrectomy, while seen in other small studies,^44^ are not corroborated by other larger studies or meta-analysis.^45^ We conducted this small evaluation in humans in order to obtain fecal samples for our mouse experiment in order to prove a causal relationship between the microbiome changes induced by bariatric surgery and NAFLD. However, our other findings in the sleeve gastrectomy cohort in regard to weight loss, improvement in NAFLD, and reduction in GIP are corroborated by larger, more diverse studies of bariatric surgery.^46,47^ Therefore, our human data add to the already established literature on bariatric surgery and microbiome changes. We also demonstrate through microbial transfer into antibiotic treated mice that changes in *Akkermansia* in the proximal small intestine were associated with changes in indolepropionate levels and GIP. However, the correlation between *Akkermansia* and metabolite changes with GIP does not provide evidence of a causal link between these factors and GIP release. Future animal studies involving *Akkermansia* and metabolite supplementation will be needed to determine any causal relationship between these factors and GIP hormone levels. Furthermore, we did not perform a full metabolome analysis of our mouse samples, therefore other potential metabolites in the serum and metabolites in the stool could also play an important role in altered GIP signaling, such as short chain fatty acids or other amino acids in addition to indolepropionate. Future studies could include full metabolomic profiles in serum and stool to examine other potential molecules that may be key in this pathway. This study shows that differences in microbiome composition caused by bariatric surgery are sufficient to alter GIP signaling, but more testing will be required to determine which bacteria-related products are responsible for that effect. Because GI hormone testing in our mice was performed, while fasting, it is also possible that other hormones were affected by the microbiome but could only be seen in a post-prandial state. Future experiments would also include repeating this same experiment in mice with an already established diagnosis of DIO and NAFLD to determine if microbiome changes are effective as treatments for DIO and NAFLD.

## Conclusion

This is the first study that we are aware of that shows that the microbiome modifies GIP signaling. This study shows that microbiome changes induced by bariatric surgery prevent diet-induced obesity and NAFLD by altering GIP signaling. These findings suggest the possibility of the microbiome as a means to prevent NAFLD development and further support the possibility of GIP antagonism as a treatment for NAFLD.

## Materials and methods

### Patient recruitment

There have been many studies to date on the effects of bariatric surgery on the gut microbiome, GI hormone signaling, and weight loss. Since our main goal was to examine the causal relationship between microbiome changes and NAFLD development, we performed a small cohort study meant to replicate other larger human bariatric cohort studies in order to collect samples for our mouse transfer experiment. Patients were recruited from the University of California, Los Angeles Bariatric Surgery Program. To avoid confounding effects of different types of surgery and potential effects of sex on the intestinal microbiome, only adult female patients undergoing laparoscopic sleeve gastrectomy were recruited. Patients were eligible if they were considering bariatric surgery, had NAFLD, and met eligibility for surgery following the Guidelines for Clinical Application of Laparoscopic Bariatric Surgery of American Gastrointestinal and Endoscopic Surgeons.^48^ NAFLD was determined by the absence of viral hepatitis, a lack of heavy alcohol use, and the presence of either elevated transaminases or imaging or pathology consistent with fatty liver disease. Patients were excluded if they had a history of major gastrointestinal surgery, cirrhosis, use of medications that affect intestinal motility, current or past alcohol or drug abuse, pregnancy, inflammatory bowel disease, irritable bowel syndrome, and use of probiotics or antibiotics within 1 month of recruitment. We collected their stools and blood before and 6 months after their surgery. Demographic information such as height, weight, race, and ethnicity was also collected. All patient research was performed in accordance with the Declaration of Helsinki and was approved by the Ethics Committee of the UCLA Institutional Review Board (IRB#13-001552).

#### Human cytokine and gastrointestinal hormone profiling

Fasting blood was collected from patients at baseline and at 6 months post-bariatric surgery. C-reactive protein (CRP) (Abcam, ab260058) and lipopolysaccharide-binding protein (LBP) (Abcam, ab279407) were tested using an Elisa kit per the manufacturer’s recommendation. In addition to hs-CRP and LBP testing, we also performed gastrointestinal hormone testing using a Luminex multiplex assay (Milliplex, Sigma) at fasting and postprandial. After a 12-hour fast, patients were given a liquid test meal (500 kcal, 70 g carbohydrate, 36 g protein, and 7 g fat). All patients finished the liquid test meal. Blood from GI hormones was measured at fasting, 30 minutes, 60 minutes, and 90 minutes postprandial. This testing was performed pre- and 6-months post-surgery. The following GI hormones were screened: amylin (total), C-peptide, GIP, GLP-1, glucagon, interleukin-6 (IL-6), insulin, leptin, MCP-1, PP, PYY, and ghrelin.

#### Human stool collection and 16S sequencing

Stool was collected within 1 week before their scheduled surgery and at 6- and 12-month post-surgery. Fresh stool was collected and frozen immediately and stored at −80°C. Afterward, frozen stool was aliquoted for further analysis. DNA was extracted using the ZymoBIOMICS DNA Microprep Kit (Zymo Research, USA) per the manufacturer’s instruction. The V4 region of the 16S ribosomal RNA gene was amplified by PCR using the 515 F-806 R primer set.^49^ Samples then underwent 250 × 2 paired-end sequencing on an Illumina HiSeq (Illumina, San Diego, CA, USA) .^49^ Raw fastq files were processed using the DADA2 pipeline in R, which assigns taxonomy using the SILVA 132 database and default parameters.^50^ After pre-processing in R utilizing DADA2, the data were incorporated into QIIME 2 version 2019.10.^51^ To remove sparse amplicon sequence variants (ASVs), ASVs were filtered if not present in at least 15% of all samples, similar to our previous publications.^9,15^ Sequence depths ranged from 60,710 to 269,258 per sample.

#### Human metabolomics

Serum was collected from patients at baseline before surgery and at 6- and 12-month post-surgery and stored at −80°C. Serum samples were then sent to Metabolon, Inc. (Morrisville, NC, USA), as a single batch on their global metabolomic and bioinformatic platform. Samples were processed and analyzed using their integrated platform that combines automated sample preparation, Liquid Chromatography/Gas Chromatography/Mass Spectrometry (LC/GC/MS), peak identification and deconvolution, and chemical intelligence.

#### Statistical analysis- human data

Demographic data such as age, BMI, and weight are expressed as means with their standard deviation. Comparison between means was done using Student’s paired *t*-test. Categorical data such as race/ethnicity were compared using Fisher's exact test. Comparison of weight, BMI, fasting glucose, hs-CRP, LBP, and GI hormone changes over time was done using a paired Wilcoxon signed-rank test.

For microbiome data, alpha diversity was calculated using the Shannon index (a metric that combines both species richness and species evenness) through QIIME2. For alpha diversity, the data were rarefied to 60,710 reads. The statistical significance of Shannon index was calculated using analysis of variance (ANOVA) adjusting for subject ID. Beta diversity was determined using the robust Aitchison distance metric in QIIME2 using the DEICODE package. This newer distance metric is better able to discriminate differences as compared to other distance metrics, such as UniFrac or Bray-Curtis.^52^ Differences in beta diversity were determined using a permutational multivariate analysis of variance through the ‘adonis’ package in R (version 4.1.2) adjusting for subject ID.^15^ Differential abundance of genera was performed using MaAsLin with patient ID as a random effect.^53^

For metabolite data, data were transformed using a median-scale normalization method in which each metabolite is divided by the median. For human data, comparison of metabolites between time points was performed using ANOVA adjusting for subject ID. *P*-values were adjusted for multiple hypothesis testing using the Benjamin-Hochberg correction method.^54^ Statistically significant metabolites were visualized using a heatmap generated in R through the ‘gplots’ package and heatmap.2 function.

#### Mouse experiment

Following an established protocol for engraftment of human microbiome into antibiotic-treated mice that utilizes a combination of systemically absorbed (ampicillin, cefoperazone, and clindamycin) and non-systemically absorbed antibiotics (ertapenem, neomycin, and vancomycin) over a 21-day course,^55^ we took samples from 4 donors (before and 6-months after surgery) and transplanted their microbiome via oral gavage into mice (C57/Bl6, males, age 6–8 weeks old) after they were treated with a 21-day course of antibiotics 3 times over a course of a week (Please see Supplemental Figure S6 for a detail description of the antibiotic treatment and gavage). The mice were then placed either on a standard double irradiated diet (10% fat by kcal, 0% fructose, and 0% cholesterol) (Research Diets, #D19082701) or a Western high-fat double-irradiated diet (40% fat, 20% fructose, 2% cholesterol) (Research Diets, #D18061301) for 90 days (4 donors × 2 time points × 2 diets × 4 mice per cage = 64 mice). The number of mice per cage is similar to other microbial transfer experiments in the past.^12^ Food was packaged into individual 1-kg bags. A new bag was used at each cage change. All mice were housed on a single rack, and only one person could change the food, water, and bedding of these mice over the 90 days to minimize cross-contamination. Please see Supplemental Figure S6 for a schematic diagram of the animal experiments. All antibiotics were given at a concentration of 1 g/L *ad libitum* in their water. About 1 g of frozen stool per timepoint and donor was resuspended in 15 ml of pre-reduced PBS and then filtered through a 100 µm filter. Mice were then given with 200 µl of this solution. Microbial concentrations of the preparation were determined using a Petroff-Hausser counting chamber, and approximately 10^10^ cells were gavage each time. All four human donors had fatty liver disease as seen on imaging or pathology before their surgery and all had significant weight loss post-surgery (at least 20% of body weight from their baseline) with resolution of their fatty liver disease seen on either imaging or pathology. This research was approved by the UCLA Animal Research Committee and the Institutional Animal Care and Use Committee.

#### Mouse body composition and glucose tolerance testing

Weight of food consumed and body weight were collected weekly. Body composition, including lean body mass and fat mass, was performed at the end of the 90 days using an EchoMRI machine (EchoMRI LLC, Houston, TX, USA). One week before the conclusion of the 90-day diet trial, mice were fasted overnight for 9 hours and given 2 g/kg of glucose intraperitoneally. Serum glucose via tail vein sampling was measured via a glucometer (Aimstrip plus, Fisher Scientific) at times 0, 30 minutes, 60 minutes, and 90 minutes.

#### Mouse sample processing

The night before euthanasia, the mice fasted overnight for 9 hours. Portal vein blood was collected by dissecting the portal vein and clamping the inferior vena cava above the liver and drawing up the blood via a capillary tube (Fischer, catalog #22260950). Because tryptophan-related metabolites are highly dependent on the microbiome^18^ and have been shown to be directly related to obesity,^19^ we focused our analysis on tryptophan-related metabolites by sending this portal vein to Metabolon, Inc. for processing as a single batch utilizing their tryptophan-related metabolite panel. Serum was then collected via heart puncture. The liver was then collected after perfusing the liver with 15 ml of sterile PBS via the inferior vena cava below the liver along with the entire gastrointestinal tract.

#### Cholesterol testing for mice

Cholesterol assay was performed using a total cholesterol-high-density lipoprotein and LDL/VLDL kit as per manufacturer’s protocol (Abcam, catalog #ab65390).

#### Liver steatosis staining and quantification

Oil red O (Abcam) staining was done on frozen OCT embedded tissue as previously described.^56^ Oil red O quantification was performed using ImageJ, averaging the percentage of areas stained across four liver sections per mouse. Hematoxylin and eosin staining were performed by the histology core at the University of California, Los Angeles. NAFLD activity scores based on hematoxylin and eosin staining were calculated by a blinded pathologist for each mouse. Triglyceride content of the liver was quantified using a calorimetric triglyceride assay kit (Abcam, ab65336).

#### Mouse GI hormone testing

Similar to human data, mouse serum was tested for circulating GI hormones and inflammatory markers using a Luminex multiplex assay (Milliplex, Sigma) after a 12-hour fast. The following GI hormones were measured: amylin (total), C-peptide, GIP, GLP-1, glucagon, IL-6, insulin, leptin, MCP-1, PP, PYY, resistin, and ghrelin (active).

#### Mouse stool collection and 16S sequencing

Fresh fecal pellets were collected on the day of euthanasia and immediately frozen and then processed for DNA and 16S sequencing similar to above with the human stool collection. Because a majority of GI hormones are released in the proximal small intestine, the proximal small intestine mucosa were collected and underwent 16S rRNA gene sequencing.

#### Statistical analysis- mouse data

Comparison between groups was performed using the Student’s *t*-test. For microbiome data, beta diversity was calculated similar to above using the robust Aitchison distance metric in QIIME2 using the DEICODE package. Differences in beta diversity were determined using a permutational multivariate analysis of variance through the ‘adonis’ package in R (version 4.1.2). To determine which bacteria were differentially abundant by diet and donor group, we ran DESeq2 in *R* which utilizes an empirical Bayesian approach to shrink dispersion and fit non-rarified data into negative binomial model.^57^ *P*-values of differential abundance testing were converted to *q*-values to correct for multiple hypothesis testing using the Benjamini–Hochberg correction.^54^ A threshold of *q* < 0.05 was used for significance. We used a similar approach to determine which bacteria were associated with hepatic triglyceride content adjusting for diet and donor groups. To evaluate the association between bacterial genera, GIP levels, and metabolites, a generalized linear model was used in R adjusting for diet and donor groups. For metabolite data, data were transformed using a median-scale normalization method similar to the above human data. Overall differences were assessed using principal component analysis. Comparison of metabolites that were independently associated with diet or donor group was performed using analysis of variance. The association of metabolite levels with GIP, bacterial candidates, and triglyceride levels was determined using a generalized linear model adjusting for diet and donor groups.

## Supplementary Material

Supplemental Material

## Data Availability

Raw sequencing data from the human cohort are available on the NIH NCBI BioProject (PRJNA885868). https://www.ncbi.nlm.nih.gov/bioproject/?term=PRJNA885868.

## References

[1] Younossi ZM, Stepanova M, Afendy M, Fang Y, Younossi Y, Mir H, Srishord M. Changes in the prevalence of the most common causes of chronic liver diseases in the United States from 1988 to 2008. Clin Gastroenterol Hepatol. 2011;9(524–30.e1):524–530.e1. doi:10.1016/j.cgh.2011.03.020. 21440669

[2] Goldberg D, Ditah IC, Saeian K, Lalehzari M, Aronsohn A, Gorospe EC, Charlton M. Changes in the prevalence of hepatitis C virus infection, nonalcoholic steatohepatitis, and alcoholic liver disease among patients with cirrhosis or liver failure on the waitlist for liver transplantation. Gastroenterology. 2017;152:1090–9 e1. doi:10.1053/j.gastro.2017.01.003. 28088461 PMC5367965

[3] Sjostrom L, Peltonen M, Jacobson P, Sjostrom CD, Karason K, Wedel H, Ahlin S, Anveden Å, Bengtsson C, Bergmark G, et al. Bariatric surgery and long-term cardiovascular events. JAMA. 2012;307:56–18. doi:10.1001/jama.2011.1914. 22215166

[4] Laursen TL, Hagemann CA, Wei C, Kazankov K, Thomsen KL, Knop FK, Grønbæk H. Bariatric surgery in patients with non-alcoholic fatty liver disease - from pathophysiology to clinical effects. World J Hepatol. 2019;11:138–149. doi:10.4254/wjh.v11.i2.138. 30820265 PMC6393715

[5] Zhu L, Baker SS, Gill C, Liu W, Alkhouri R, Baker RD, Gill SR. Characterization of gut microbiomes in nonalcoholic steatohepatitis (NASH) patients: a connection between endogenous alcohol and NASH. Hepatology (Baltimore, Md). 2013;57:601–609. doi:10.1002/hep.26093. 23055155

[6] Louis S, Tappu RM, Damms-Machado A, Huson DH, Bischoff SC, Covasa M. Characterization of the gut microbial community of obese patients following a weight-loss intervention using whole metagenome shotgun sequencing. PLoS One. 2016;11:e0149564. doi:10.1371/journal.pone.0149564. 26919743 PMC4769288

[7] Bajaj JS, Hylemon PB, Ridlon JM, Heuman DM, Daita K, White MB, Monteith P, Noble NA, Sikaroodi M, Gillevet PM, et al. Colonic mucosal microbiome differs from stool microbiome in cirrhosis and hepatic encephalopathy and is linked to cognition and inflammation. Am J Physiol Gastrointest Liver Physiol. 2012;303:G675–85. doi:10.1152/ajpgi.00152.2012. 22821944 PMC3468538

[8] Turnbaugh PJ, Hamady M, Yatsunenko T, Cantarel BL, Duncan A, Ley RE, Sogin ML, Jones WJ, Roe BA, Affourtit JP, et al. A core gut microbiome in obese and lean twins. Nature. 2009;457:480–484. doi:10.1038/nature07540. 19043404 PMC2677729

[9] Dong TS, Katzka W, Lagishetty V, Luu K, Hauer M, Pisegna J, Jacobs JP. A microbial signature identifies advanced fibrosis in patients with chronic liver disease mainly due to NAFLD. Sci Rep. 2020;10:2771. doi:10.1038/s41598-020-59535-w. 32066758 PMC7026172

[10] Million M, Lagier JC, Yahav D, Paul M. Gut bacterial microbiota and obesity. Clin Microbiol Infect. 2013;19:305–313. doi:10.1111/1469-0691.12172. 23452229

[11] Turnbaugh PJ, Ridaura VK, Faith JJ, Rey FE, Knight R, Gordon JI. The effect of diet on the human gut microbiome: a metagenomic analysis in humanized gnotobiotic mice. Sci Trans Med. 2009;1:6ra14–6ra. doi:10.1126/scitranslmed.3000322. PMC2894525 20368178

[12] Tremaroli V, Karlsson F, Werling M, Ståhlman M, Kovatcheva-Datchary P, Olbers T. Roux-en-Y gastric bypass and vertical banded gastroplasty induce long-term changes on the human gut microbiome contributing to fat mass regulation. Cell Metab. 2015;22:228–238. doi:10.1016/j.cmet.2015.07.009. 26244932 PMC4537510

[13] Dong TS, Luu K, Lagishetty V, Sedighian F, Woo S-L, Dreskin BW. Gut microbiome profiles associated with steatosis severity in metabolic associated fatty liver disease. Hepatoma Res. 2021;7:37. 10.20517/2394-5079.2021.55 PMC9881202 36713356

[14] Dong TS, Jacobs JP. Nonalcoholic fatty liver disease and the gut microbiome: are bacteria responsible for fatty liver? Experimental biology and medicine (Maywood). 2019;244(6):408–418. doi:10.1177/1535370219836739. 30871368 PMC6547005

[15] Dong TS, Luu K, Lagishetty V, Sedighian F, Woo SL, Dreskin BW, Katzka W, Chang C, Zhou Y, Arias-Jayo N, et al. The intestinal microbiome predicts weight loss on a calorie-restricted diet and is associated with improved hepatic steatosis. Front Nutr. 2021;8:718661. doi:10.3389/fnut.2021.718661. 34307440 PMC8295485

[16] Thondam SK, Cuthbertson DJ, Wilding JPH. The influence of Glucose-dependent Insulinotropic Polypeptide (GIP) on human adipose tissue and fat metabolism: implications for obesity, type 2 diabetes and Non-Alcoholic Fatty Liver Disease (NAFLD). Peptides. 2020;125:170208. doi:10.1016/j.peptides.2019.170208. 31759125

[17] Hansen CH, Nielsen DS, Kverka M, Zakostelska Z, Klimesova K, Hudcovic T, Tlaskalova-Hogenova H, Hansen AK. Patterns of early gut colonization shape future immune responses of the host. PLoS One. 2012;7:e34043. doi:10.1371/journal.pone.0034043. 22479515 PMC3313961

[18] Wikoff WR, Anfora AT, Liu J, Schultz PG, Lesley SA, Peters EC, Siuzdak G. Metabolomics analysis reveals large effects of gut microflora on mammalian blood metabolites. Proc Natl Acad Sci U S A. 2009;106:3698–3703. doi:10.1073/pnas.0812874106. 19234110 PMC2656143

[19] Dong TS, Mayer EA, Osadchiy V, Chang C, Katzka W, Lagishetty V, Gonzalez K, Kalani A, Stains J, Jacobs JP, et al. A distinct brain-gut-microbiome profile exists for females with obesity and food addiction. Obesity (Silver Spring). 2020;28:1477–1486. doi:10.1002/oby.22870. 32935533 PMC7494955

[20] Viana EC, Araujo-Dasilio KL, Miguel GP, Bressan J, Lemos EM, Moyses MR, et al. Gastric bypass and sleeve gastrectomy: the same impact on IL-6 and TNF-alpha. Prospective Clinical Trial Obes Surg. 2013;23:1252–1261. 10.1007/s11695-013-0894-2 23475776

[21] Gletsu N, Lin E, Zhu JL, Khaitan L, Ramshaw BJ, Farmer PK, Ziegler TR, Papanicolaou DA, Smith CD. Increased plasma interleukin 6 concentrations and exaggerated adipose tissue interleukin 6 content in severely obese patients after operative trauma. Surgery. 2006;140:50–57. doi:10.1016/j.surg.2006.01.018. 16857442

[22] Catalan V, Gomez-Ambrosi J, Rodriguez A, Ramirez B, Rotellar F, Valenti V, Silva C, Gil MJ, Salvador J, Frühbeck G, et al. Increased levels of chemerin and its receptor, chemokine-like receptor-1, in obesity are related to inflammation: tumor necrosis factor-alpha stimulates mRNA levels of chemerin in visceral adipocytes from obese patients. Surg Obes Relat Dis. 2013;9:306–314. doi:10.1016/j.soard.2011.11.001. 22154272

[23] Myers MG Jr., Leibel RL, Seeley RJ, Schwartz MW. Obesity and leptin resistance: distinguishing cause from effect. Trends Endocrinol Metab. 2010;21:643–651. doi:10.1016/j.tem.2010.08.002. 20846876 PMC2967652

[24] Cornejo-Pareja I, Munoz-Garach A, Clemente-Postigo M, Tinahones FJ. Importance of gut microbiota in obesity. Eur J Clin Nutr. 2019;72:26–37. doi:10.1038/s41430-018-0306-8. 30487562

[25] Clarke SF, Murphy EF, Nilaweera K, Ross PR, Shanahan F, O’Toole PW, Cotter PD. The gut microbiota and its relationship to diet and obesity: new insights. Gut Microbes. 2012;3:186–202. doi:10.4161/gmic.20168. 22572830 PMC3427212

[26] Baothman OA, Zamzami MA, Taher I, Abubaker J, Abu-Farha M. The role of gut microbiota in the development of obesity and Diabetes. Lipids Health Dis. 2016;15:108. doi:10.1186/s12944-016-0278-4. 27317359 PMC4912704

[27] Bauer PV, Hamr SC, Duca FA. Regulation of energy balance by a gut-brain axis and involvement of the gut microbiota. Cmls. 2016;73:737–755. doi:10.1007/s00018-015-2083-z. 26542800 PMC11108299

[28] Maruvada P, Leone V, Kaplan LM, Chang EB. The human microbiome and obesity: moving beyond associations. Cell Host Microbe. 2017;22:589–599. doi:10.1016/j.chom.2017.10.005. 29120742

[29] Miyawaki K, Yamada Y, Ban N, Ihara Y, Tsukiyama K, Zhou H, Fujimoto S, Oku A, Tsuda K, Toyokuni S, et al. Inhibition of gastric inhibitory polypeptide signaling prevents obesity. Nat Med. 2002;8:738–742. doi:10.1038/nm727. 12068290

[30] Joo E, Harada N, Yamane S, Fukushima T, Taura D, Iwasaki K, Sankoda A, Shibue K, Harada T, Suzuki K, et al. Inhibition of gastric inhibitory polypeptide receptor signaling in adipose tissue reduces insulin resistance and hepatic steatosis in high-fat diet-fed mice. Diabetes. 2017;66:868–879. doi:10.2337/db16-0758. 28096257

[31] Pfeiffer AFH, High Glycemic K-NF. Index metabolic damage - a pivotal role of GIP and GLP-1. Trends Endocrinol Metab. 2018;29:289–299. doi:10.1016/j.tem.2018.03.003. 29602522

[32] Keyhani-Nejad F, Irmler M, Isken F, Wirth EK, Beckers J, Birkenfeld AL, Pfeiffer AFH. Nutritional strategy to prevent fatty liver and insulin resistance independent of obesity by reducing glucose-dependent insulinotropic polypeptide responses in mice. Diabetologia. 2015;58:374–383. doi:10.1007/s00125-014-3423-5. 25348610

[33] Killion EA, Chen M, Falsey JR, Sivits G, Hager T, Atangan L, Helmering J, Lee J, Li H, Wu B, et al. Chronic glucose-dependent insulinotropic polypeptide receptor (GIPR) agonism desensitizes adipocyte GIPR activity mimicking functional GIPR antagonism. Nat Commun. 2020;11:4981. doi:10.1038/s41467-020-18751-8. 33020469 PMC7536395

[34] McClean PL, Irwin N, Cassidy RS, Holst JJ, Gault VA, Flatt PR. GIP receptor antagonism reverses obesity, insulin resistance, and associated metabolic disturbances induced in mice by prolonged consumption of high-fat diet. Am J Physiol Endocrinol Metab. 2007;293:E1746–55. doi:10.1152/ajpendo.00460.2007. 17848629

[35] Shin N-R, Lee J-C, Lee H-Y, Kim M-S, Whon TW, Lee M-S, et al. An increase in the Akkermansia spp Population Induced by Metformin Treatment Improves Glucose Homeostasis in diet-induced Obese Mice Gut. 2014;63:727–735. 10.1136/gutjnl-2012-303839 23804561

[36] Schneeberger M, Everard A, Gómez-Valadés AG, Matamoros S, Ramírez S, Delzenne NM, Gomis R, Claret M, Cani PD. Akkermansia muciniphila inversely correlates with the onset of inflammation, altered adipose tissue metabolism and metabolic disorders during obesity in mice. Sci Rep. 2015;5:16643. doi:10.1038/srep16643. 26563823 PMC4643218

[37] Macchione IG, Lopetuso LR, Ianiro G, Napoli M, Gibiino G, Rizzatti G, Petito V, Gasbarrini A, Scaldaferri F. Akkermansia muciniphila: key player in metabolic and gastrointestinal disorders. Eur Rev Med Pharmacol Sci. 2019;23:8075–8083. doi:10.26355/eurrev_201909_19024. 31599433

[38] Thingholm LB, Ruhlemann MC, Koch M, Fuqua B, Laucke G, Boehm R, Bang C, Franzosa EA, Hübenthal M, Rahnavard A, et al. Obese Individuals with and without type 2 diabetes show different gut microbial functional capacity and composition. Cell Host Microbe. 2019;26:252–64 e10. doi:10.1016/j.chom.2019.07.004. 31399369 PMC7720933

[39] Dao MC, Everard A, Aron-Wisnewsky J, Sokolovska N, Prifti E, Verger EO, Kayser BD, Levenez F, Chilloux J, Hoyles L, et al. Akkermansia muciniphila and improved metabolic health during a dietary intervention in obesity: relationship with gut microbiome richness and ecology. Gut. 2016;65:426–436. doi:10.1136/gutjnl-2014-308778. 26100928

[40] Xu Y, Wang N, Tan HY, Li S, Zhang C, Feng Y. Function of akkermansia muciniphila in obesity: interactions with lipid metabolism, immune response and gut systems. Front Microbiol. 2020;11:219. doi:10.3389/fmicb.2020.00219. 32153527 PMC7046546

[41] Depommier C, Everard A, Druart C, Plovier H, Van Hul M, Vieira-Silva S, Falony G, Raes J, Maiter D, Delzenne NM, et al. Supplementation with Akkermansia muciniphila in overweight and obese human volunteers: a proof-of-concept exploratory study. Nat Med. 2019;25:1096–1103. doi:10.1038/s41591-019-0495-2. 31263284 PMC6699990

[42] Venkatesh M, Mukherjee S, Wang H, Li H, Sun K, Benechet AP, Qiu Z, Maher L, Redinbo M, Phillips R, et al. Symbiotic bacterial metabolites regulate gastrointestinal barrier function via the xenobiotic sensor PXR and toll-like receptor 4. Immunity. 2014;41:296–310. doi:10.1016/j.immuni.2014.06.014. 25065623 PMC4142105

[43] de Mello VD, Paananen J, Lindström J, Lankinen MA, Shi L, Kuusisto J, Pihlajamäki J, Auriola S, Lehtonen M, Rolandsson O, et al. Indolepropionic acid and novel lipid metabolites are associated with a lower risk of type 2 diabetes in the Finnish diabetes prevention study. Sci Rep. 2017;7:46337. doi:10.1038/srep46337. 28397877 PMC5387722

[44] Nannipieri M, Baldi S, Mari A, Colligiani D, Guarino D, Camastra S, Barsotti E, Berta R, Moriconi D, Bellini R, et al. Roux-en-Y gastric bypass and sleeve gastrectomy: mechanisms of diabetes remission and role of gut hormones. J Clin Endocrinol Metab. 2013;98:4391–4399. doi:10.1210/jc.2013-2538. 24057293

[45] Ionut V, Burch M, Youdim A, Bergman RN. Gastrointestinal hormones and bariatric surgery-induced weight loss. Obesity (Silver Spring). 2013;21:1093–1103. doi:10.1002/oby.20364. 23512841 PMC4423817

[46] Fakhry TK, Mhaskar R, Schwitalla T, Muradova E, Gonzalvo JP, Murr MM. Bariatric surgery improves nonalcoholic fatty liver disease: a contemporary systematic review and meta-analysis. Surg Obes Relat Dis. 2019;15:502–511. doi:10.1016/j.soard.2018.12.002. 30683512

[47] Rao RS, Kini S. GIP and bariatric surgery. Obes Surg. 2011;21:244–252. doi:10.1007/s11695-010-0305-x. 21082290

[48] Farrell TM, Haggerty SP, Overby DW, Kohn GP, Richardson WS, Fanelli RD. Clinical application of laparoscopic bariatric surgery: an evidence-based review. Surg Endosc. 2009;23:930–949. doi:10.1007/s00464-008-0217-1. 19125308

[49] Tong M, Jacobs JP, Mchardy IH, Braun J, Angeles L. Sampling of intestinal microbiota and targeted amplification of bacterial 16S rRNA genes for microbial ecologic analysis. Curr Proto Immun. 2014;107:7.41.1–11. 10.1002/0471142735.im0741s107 PMC4457454 25367129

[50] Callahan BJ, McMurdie PJ, Rosen MJ, Han AW, Johnson AJA, Holmes SP. DADA2: high-resolution sample inference from Illumina amplicon data. Nat Methods. 2016;13:581–583. doi:10.1038/nmeth.3869. 27214047 PMC4927377

[51] Bolyen E, Rideout JR, Dillon MR, Bokulich NA, Abnet CC, Al-Ghalith GA, Alexander H, Alm EJ, Arumugam M, Asnicar F, et al. Reproducible, interactive, scalable and extensible microbiome data science using QIIME 2. Nat Biotechnol. 2019;37:852–857. doi:10.1038/s41587-019-0209-9. 31341288 PMC7015180

[52] Martino C, Morton JT, Marotz CA, Thompson LR, Tripathi A, Knight R, Zengler K. A novel sparse compositional technique reveals microbial perturbations. mSystems. 2019;4:e00016–19. doi:10.1128/mSystems.00016-19. 30801021 PMC6372836

[53] Morgan XC, Tickle TL, Sokol H, Gevers D, Devaney KL, Ward DV, Reyes JA, Shah SA, LeLeiko N, Snapper SB, et al. Dysfunction of the intestinal microbiome in inflammatory bowel disease and treatment. Genome Biol. 2012;13:R79. doi:10.1186/gb-2012-13-9-r79. 23013615 PMC3506950

[54] Benjamini Y, Hochberg Y. Controlling the false discovery rate: a practical and powerful approach to multiple testing. J Royal Stat Soc Series B (Methodological). 1995;57:289–300. doi:10.1111/j.2517-6161.1995.tb02031.x.

[55] Staley C, Kaiser T, Beura LK, Hamilton MJ, Weingarden AR, Bobr A, Kang J, Masopust D, Sadowsky MJ, Khoruts A, et al. Stable engraftment of human microbiota into mice with a single oral gavage following antibiotic conditioning. Microbiome. 2017;5:87. doi:10.1186/s40168-017-0306-2. 28760163 PMC5537947

[56] Mehlem A, Hagberg CE, Muhl L, Eriksson U, Falkevall A. Imaging of neutral lipids by oil red O for analyzing the metabolic status in health and disease. Nat Protoc. 2013;8:1149–1154. doi:10.1038/nprot.2013.055. 23702831

[57] Love MI, Huber W, Anders S. Moderated estimation of fold change and dispersion for RNA-seq data with DESeq2. Genome Biol. 2014;15:550. doi:10.1186/s13059-014-0550-8. 25516281 PMC4302049

