# Supplementary material for: Microbial changes from bariatric surgery alters glucose-dependent insulinotropic polypeptide and prevents fatty liver disease
Source: Gut Microbes. 2023 Feb 2;15(1):2167170. doi: 10.1080/19490976.2023.2167170 (PMC9897796; doi:10.1080/19490976.2023.2167170)
Supplement: Supplemental Material [file KGMI_A_2167170_SM8569.docx]

| Average (SD) (n=18) | Pre-Surgery | Post-Surgery (6 mo) | p-value |
| --- | --- | --- | --- |
| Age (yr) | 37.1 (9.4) | 37.1 (9.4) | NA |
| BMI | 44.7 (4.9) | 33.9 (4.8) | <0.001 |
| Weight (kg) | 118.5 (18.8) | 89.7 (16.9) | <0.001 |
| Race/Ethnicity | | | |
| Non-Hispanic White (%) | 44.4 |  | NA |
| African American (%) | 5.6 |  |  |
| Asian (%) | 11.1 |  |  |
| Hispanic (%) | 38.9 |  |  |

**Supplemental Table S1:** Patient demographic of sleeve gastrectomy cohort

| Donor | Race/Ethnicity | Age | Pre-Surgery BMI | Post-Surgery BMI | % Body Weight Loss at 6 Month | Fatty Liver Resolution on Imaging |
| --- | --- | --- | --- | --- | --- | --- |
| A | Non-Hispanic White | 25 | 51.0 | 37.1 | 20.1 | Yes |
| B | Hispanic | 21 | 42.2 | 30.9 | 23.3 | Yes |
| C | Asian | 45 | 36.9 | 25.1 | 35.3 | Yes |
| D | Hispanic | 43 | 38.2 | 31.0 | 20.2 | Yes |

**Supplemental Table S2:** Demographic information of the 4 donors used for mouse experiment

| kynurenine |
| --- |
| 3-indoxyl sulfate |
| N-formylanthranilic acid |
| anthranilate |
| oxindolylalanine |
| picolinate |
| N-acetylkynurenine (2) |
| indole-3-carboxylate |
| indolepropionate |
| indoxyl glucuronide |
| N-acetyltryptophan |
| C-glycosyltryptophan |
| indoleacrylate |
| indoleacetate |
| kynurenate |
| serotonin |
| indolelactate |
| indoleacetylglycine |
| tryptophan |
| xanthurenate |
| 5-hydroxyindoleacetate |

**Supplemental Table S3:** Tryptophan-related metabolites tested


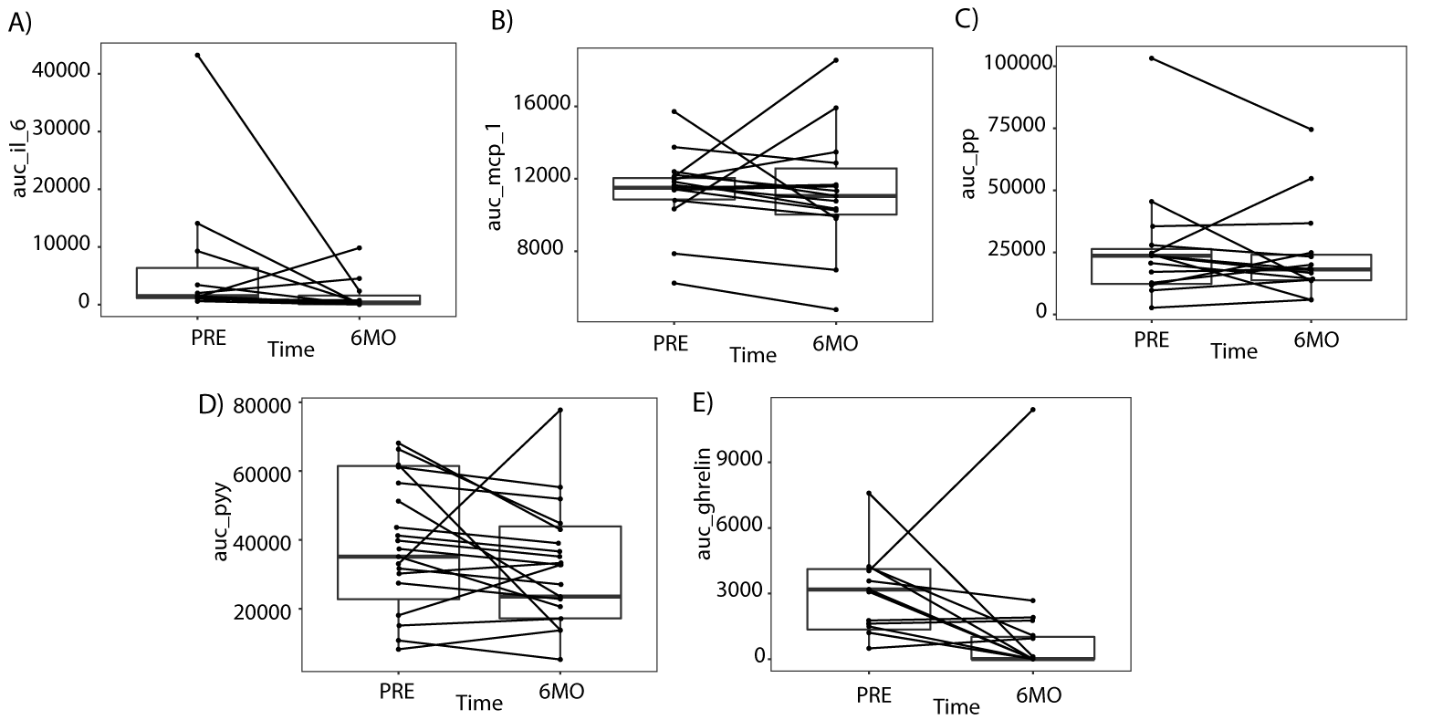


**Supplemental Figure S1**: Gastrointestinal hormones of human patients before and after sleeve gastrectomy. For the GI hormones, patients were given a standard meal and blood at fasting, 30 minutes, 60 minutes, and 90 minutes were sampled for various GI hormones. Circulating GI hormone was then measured across time and the area under the curve (AUC) for each hormone at baseline and at 6-months post-sleeve gastrectomy is shown for A) IL-6, B) monocyte chemoattractant protein 1 (MCP-1), C) pancreatic polypeptide (PP), D) peptide YY (PYY), and E) ghrelin. All these hormones were not significantly different by timepoint.


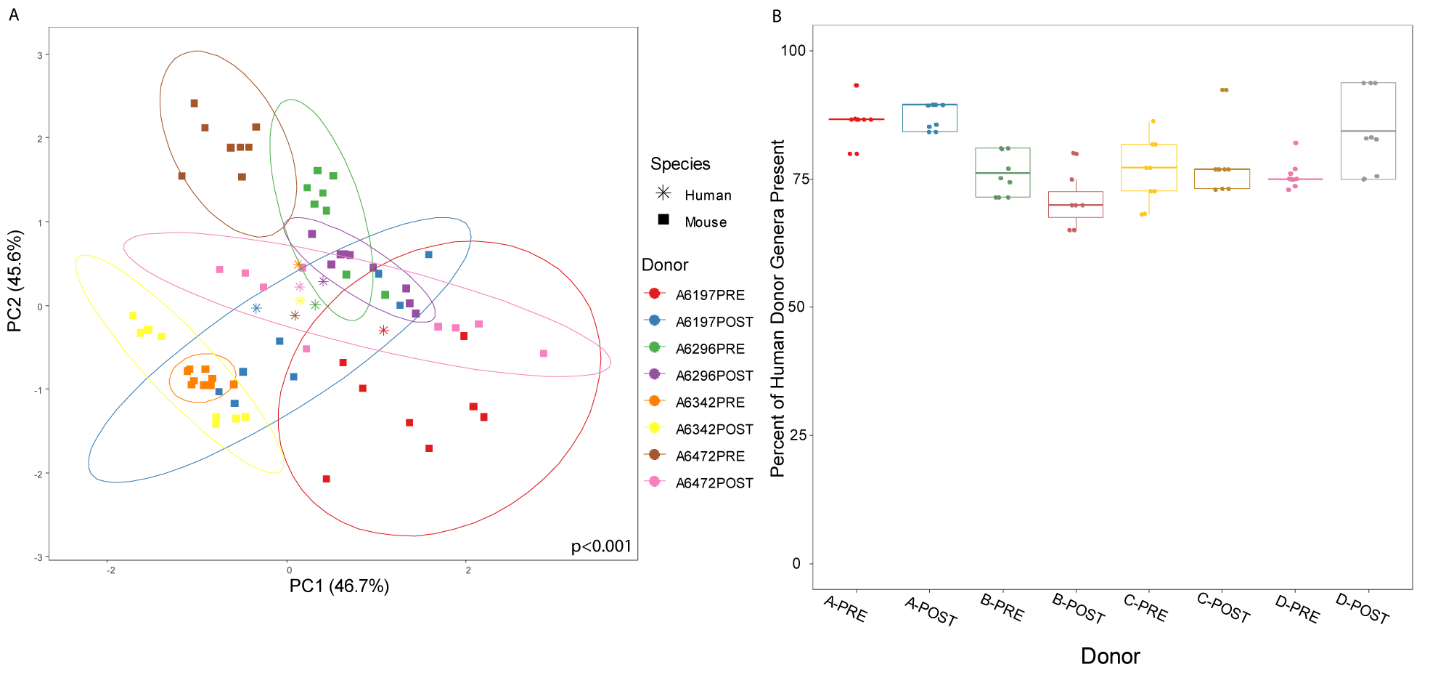


**Supplemental Figure S2**: A) Principal coordinate analysis plot (i.e beta diversity) of mice stool samples with their respective donors. Ellipses shown are 95% confidence interval. B) Percent of human donor genera present in mouse fecal samples by donor.


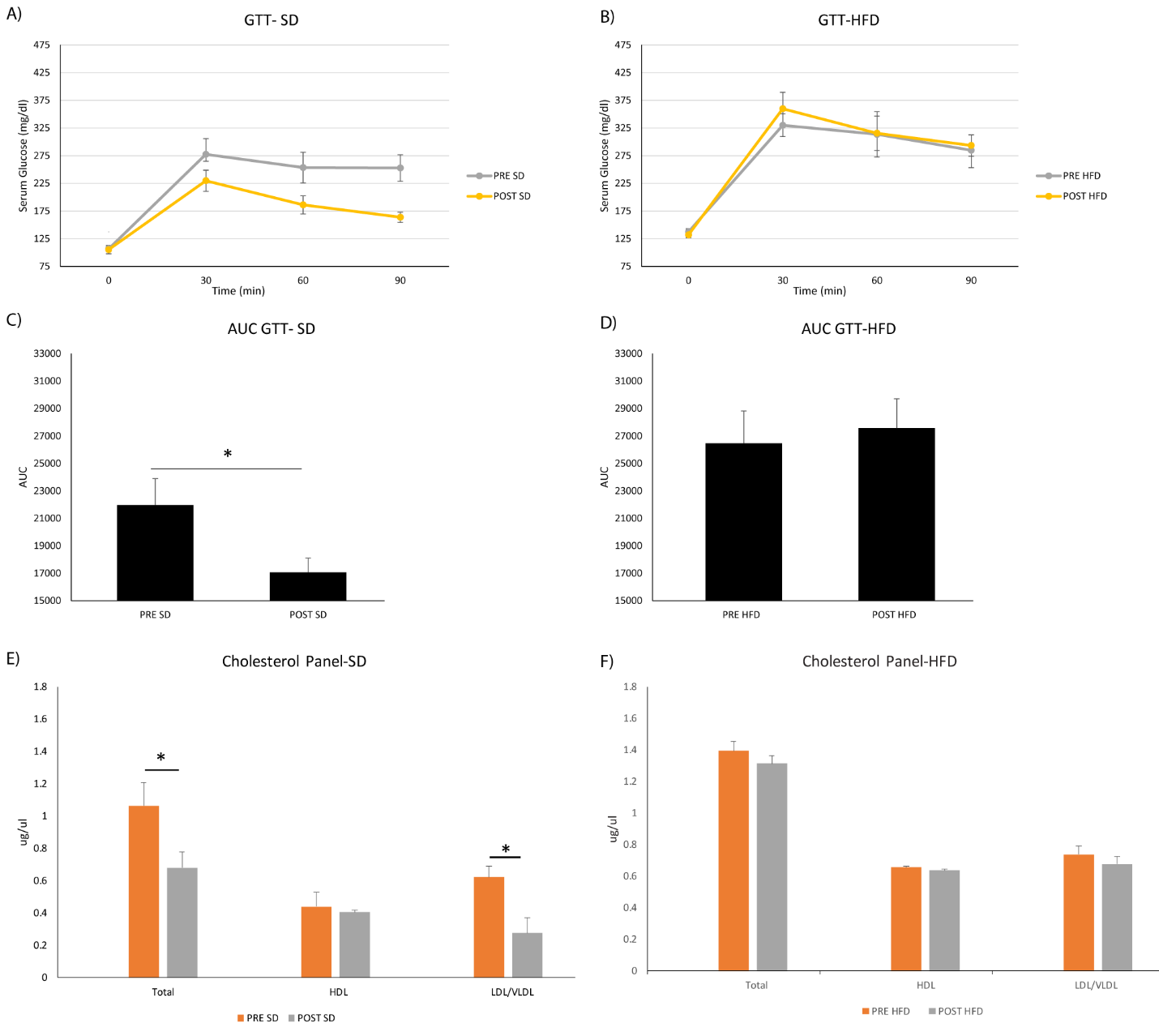


**Supplemental Figure S3**: Glucose tolerance testing result of mice with different microbiome donors (PRE vs POST) while on a A) standard diet (SD) or a B) high fat, high fructose, high cholesterol diet (HFD). C,D) Area under the curve (AUC) of the respective graphs for A) and B). Cholesterol profile of mice on a E) SD or a F) HFD. *indicates p-value<0.05.


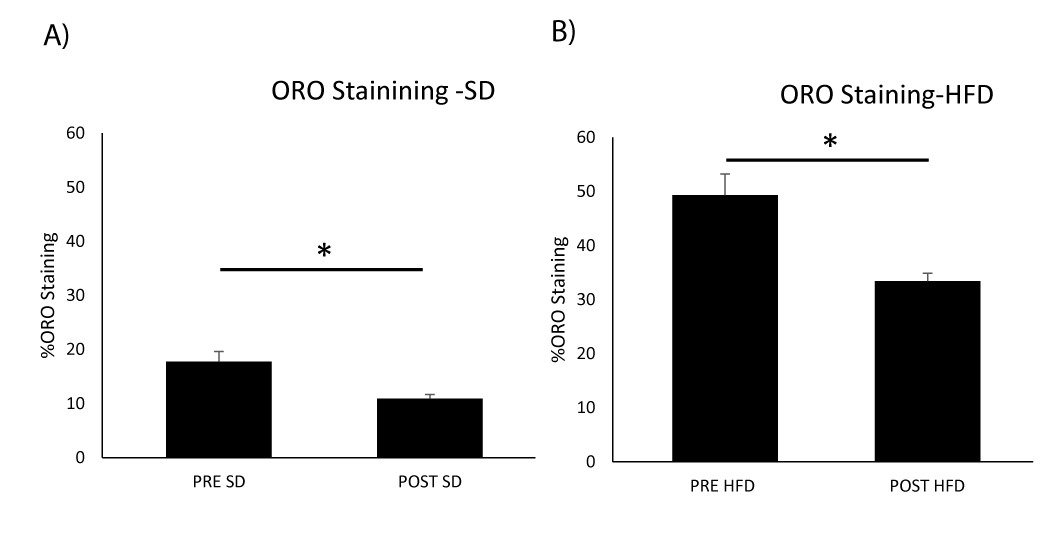


**Supplemental Figure S4**: Oil red o quantification by ImageJ of mice livers on a A) standard diet (SD) or a B) high fat, high fructose, high cholesterol diet (HFD). *indicates p-value <0.05


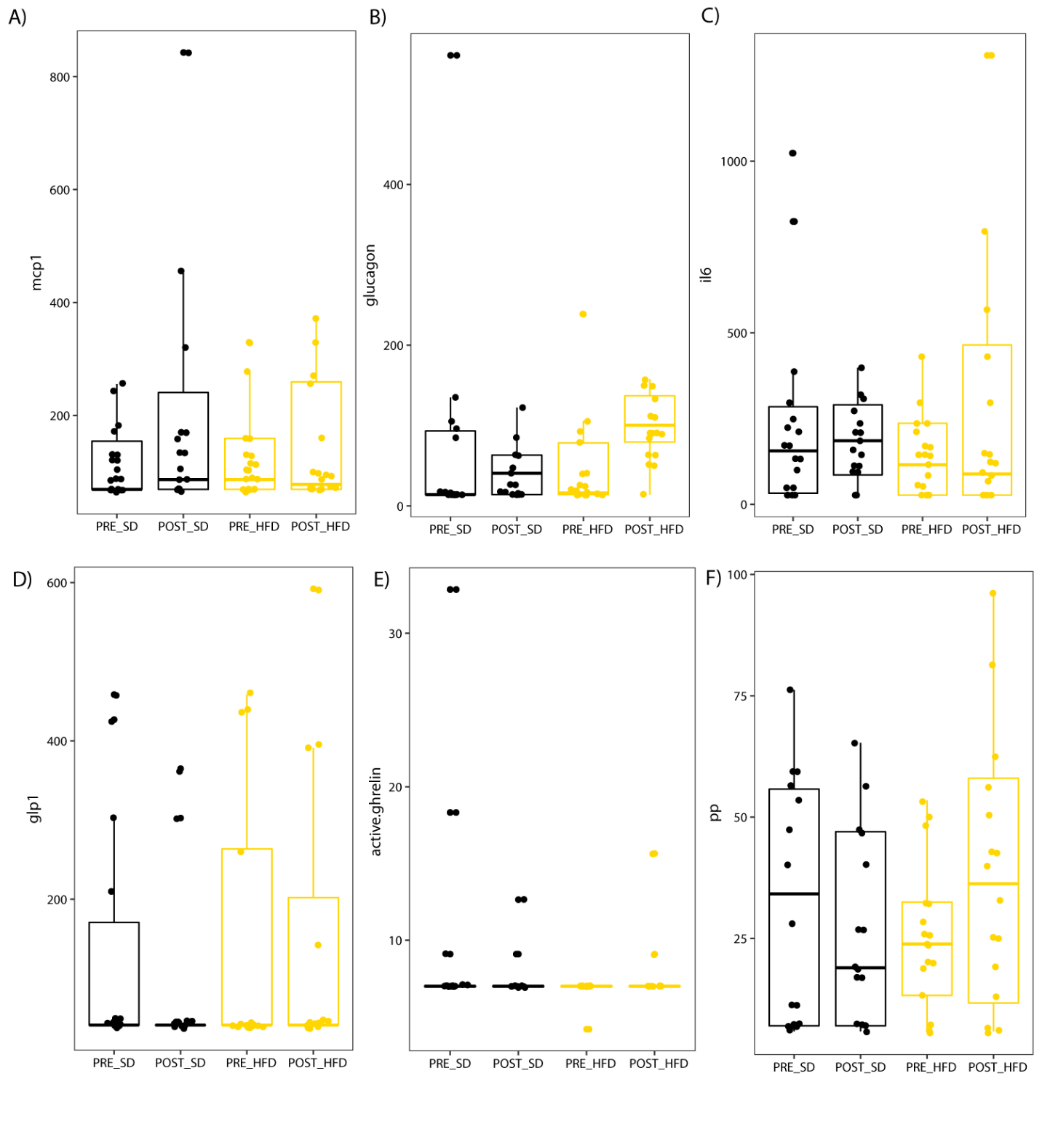


**Supplemental Figure S5**: Serum samples of mice were collected after a 9 hour fast. Hormone profile is shown for A) MCP-1, B) glucagon, C) IL6, D) GLP-1, E) ghrelin (active), and PP. Colored by diet (SD vs HFD) and by donor group (PRE vs POST). No significant differences were seen either by diet or donor group of any hormones listed. Each scale is pg/ml.


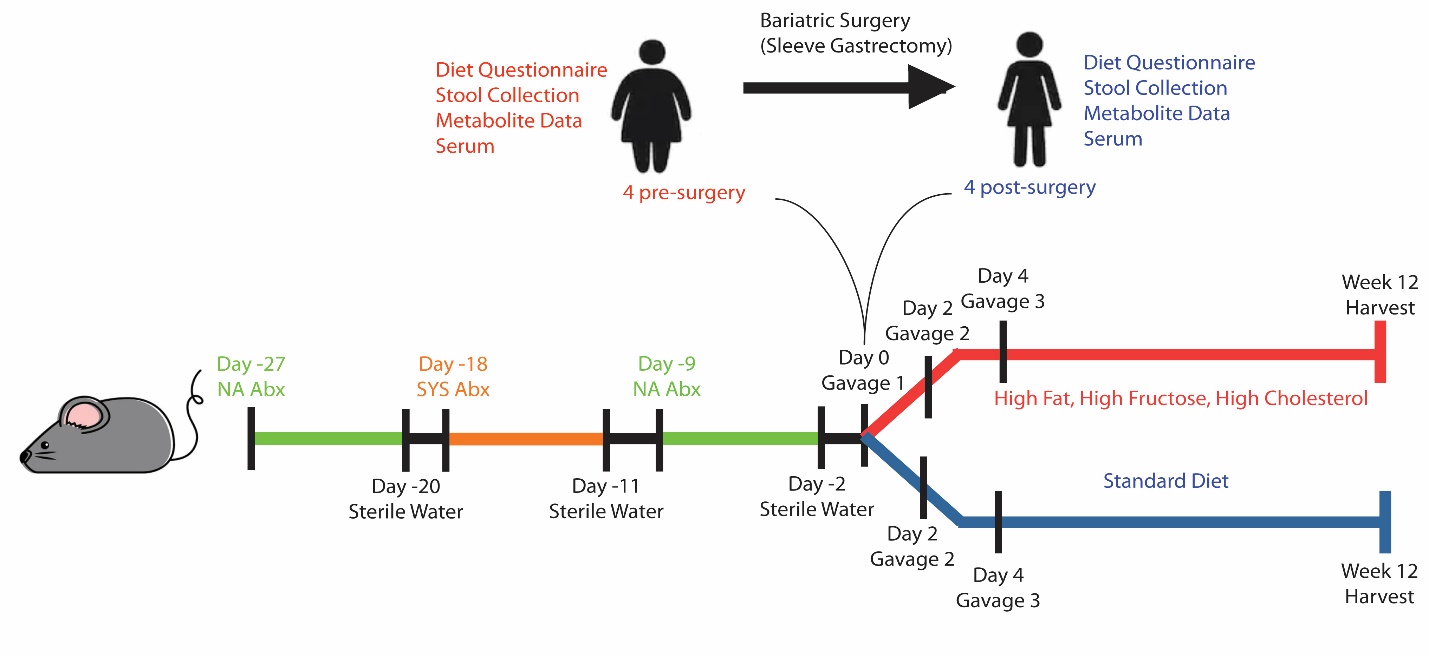


**Supplemental Figure S6:** Study design for mice microbiota engraftment
